# Chlorin e6 Conjugated Interleukin-6 Receptor Aptamers Selectively Kill Target Cells Upon Irradiation

**DOI:** 10.1038/mtna.2013.70

**Published:** 2014-01-21

**Authors:** Sven Kruspe, Cindy Meyer, Ulrich Hahn

**Affiliations:** 1 Chemistry Department, MIN-Faculty, Institute for Biochemistry and Molecular Biology, Hamburg University, Hamburg, Germany; 2 Current address: Howard Hughes Medical Institute, Laboratory of RNA Molecular Biology, The Rockefeller University, New York, USA

**Keywords:** aptamers, human interleukin-6 receptor, photodynamic therapy, receptor mediated endocytosis

## Abstract

Photodynamic therapy (PDT) uses the therapeutic properties of light in combination with certain chemicals, called photosensitizers, to successfully treat brain, breast, prostate, and skin cancers. To improve PDT, current research focuses on the development of photosensitizers to specifically target cancer cells. In the past few years, aptamers have been developed to directly deliver cargo molecules into target cells. We conjugated the photosensitizer chlorin e6 (ce6) with a human interleukin-6 receptor (IL-6R) binding RNA aptamer, AIR-3A yielding AIR-3A-ce6 for application in high efficient PDT. AIR-3A-ce6 was rapidly and specifically internalized by IL-6R presenting (IL-6R^+^) cells. Upon light irradiation, targeted cells were selectively killed, while free ce6 did not show any toxic effect. Cells lacking the IL-6R were also not affected by AIR-3A-ce6. With this approach, we improved the target specificity of ce6-mediated PDT. In the future, other tumor-specific aptamers might be used to selectively localize photosensitizers into cells of interest and improve the efficacy and specificity of PDT in cancer and other diseases.

## Introduction

### Photodynamic therapeutic agents

Photodynamic therapy (PDT) is clinically used as a nonsurgical treatment option for several diseases, such as malignant cancers. PDT uses photosensitizing agents, called photosensitizers, that accumulate more or less selectively in target cells.^1,2^ Photosensitizers, such as chlorins (*e.g.*, Tomoporphin sold as Foscan),^3,4^ are excited from the singlet electron ground state to a higher singlet state upon illumination with light sources of the appropriate wavelength, typically 600–800 nm. Subsequently, this leads to the generation of cytotoxic compounds, like singlet oxygen (^1^O_2_) and reactive oxygen species, which could result in proliferation decline, induced cell cycle arrest, apoptosis or necrotic cell death of affected cells.^5^ PDT, therefore, is a feasible strategy for solid tumor therapy. One drawback of PDT is its lack of specificity for certain cell types, leading to adverse side effects in surrounding tissue.

### AIR-3A: an IL-6 receptor–specific RNA aptamer

Aptamers, small oligonucleotides with high affinity and specificity for target molecules, are comparable to antibodies regarding their recognition properties. In addition, aptamers possess remarkable advantages, such as little to no immunogenicity and toxicity, longer shelf-life, and lower production costs. In the last two decades, aptamers have been selected for a huge variety of target molecules, including ions,^6^ fluorescent dyes,^7^ antibiotics,^8^ peptides,^9^ and proteins.^10^ Furthermore, aptamers targeting viruses^11^ as well as whole cells^12^ have been previously selected and characterized for potential therapeutic applications.

Usually, aptamers, as polyanions, are not able to pass cellular membranes. If, however, aptamers do specifically bind receptors presented on cell surfaces, cells might be able to take them up in tandem with the receptor via a naturally occurring internalization process called receptor-mediated endocytosis (RME).^13,14^ We recently described the interleukin-6 receptor (IL-6R)–specific RNA aptamer (AIR-3A), which is internalized by cells through RME.^15^ We were able to visualize AIR-3A–mediated internalization of fluorescently labeled streptavidin by IL-6R presenting cells. However, the intracellular traffic of the cargo subsequent to RME remains a challenge for therapeutic effects.^16^ Since most of the RME pathways lead to acidification in the endosome and recycling or lysosomal degradation of the receptor and its ligand,^17^ therapeutic cargo molecules remain ineffective as they are degraded or trapped within the endosomal vesicle. Thus, therapeutics that benefit from acidification to undergo an endosomal escape are of great interest.

IL-6 is a multifunctional cytokine that is involved in many immune and inflammatory responses.^18^ The multifunctional cytokine IL-6 and its receptors are involved in the progression of various inflammatory diseases, such as Crohn's disease, rheumatoid arthritis, and certain cancers, such as multiple myeloma or hepatocellular carcinoma.^19^ IL-6 and IL-6R are also associated to skin diseases including psoriasis and systemic sclerosis.^20^ Therefore, a promising therapeutic strategy to tackle the aforementioned diseases might be to specifically deliver drugs into cells by selectively targeting IL-6R.

The 19 nucleotide, short RNA aptamer, AIR-3A, binds with high affinity (*K*_d_ = 8.5 nmol/l) and specificity to human IL-6R presenting cells. Due to RME, this aptamer serves as a specific drug delivery transporter. Here, we report AIR-3A as a specific and efficient delivery system for the PDT agent chlorin e6 into IL-6R presenting cells and the subsequent induction of cell death upon irradiation. Our approach paves the way for a more specific PDT enabled by the application of a highly specific aptamer.

## Results

### Construction of the chlorin e6 aptamer conjugate AIR-3A-ce6

To conjugate the RNA aptamer AIR-3A and the photosensitizer ce6, we directly linked an aminomodified version of AIR-3A to ce6 through an EDC-/NHS-mediated amine coupling as outlined in the Materials and Methods. After removal of unreacted ce6, the modified AIR-3A showed a reduced electrophoretic mobility in comparison to the educt oligonucleotide (**Supplementary Figure S1**) demonstrating a high coupling efficiency. The migration shift (~2 nt) was in accordance with the mass of ce6. As a negative control, we used ce6 coupled to a shuffled version of AIR-3A that was not able to bind IL-6R.

### Cellular binding of AIR-3A-ce6

**Figure 1:**
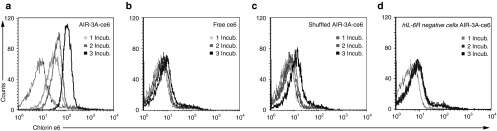
**Detection of chlorin e6 in BaF3/gp130/IL6R/TNF cells**. Cells were incubated three times with chlorin e6 derivatized aptamer (AIR-3A-ce6, 50 nmol/l) for 45 minutes in media without serum. Between these, incubations cells were cultivated in media containing serum for 5 hours each. Internalization was analyzed via flow cytometry for cells treated with (**a**) AIR-3A-ce6, (**b**) free chlorin e6, (**c**) shuffled-AIR-3A-ce6, (**d**) and hIL-6R- cells as control.

We analyzed the binding ability of AIR-3A-ce6 to BaF3/gp130/IL6R/TNF cells—murine cells presenting human IL-6 receptor (hIL-6R) shown by flow cytometry. Cells incubated with AIR-3A-ce6 (50 nmol/l) exhibited enhanced ce6 fluorescence after an exposure of 45 minutes compared to cells incubated with free ce6 (**Figure 1a**). After an additional second incubation cycle, the fluorescence signal of the cells increased whereas the same treatment with free ce6 had not such an effect (**Figure 1b**). Incubation of hIL-6R^+^ cells with 50 nmol/l of shuffled-AIR-3A-ce6 conjugate also resulted in nonfluorescing cells (**Figure 1c**). When hIL-6R-lacking cells were exposed to AIR-3A-ce6, only slight nonspecific binding to the cell surface could be detected (**Figure 1d**).

### Uptake of AIR-3A-ce6 by hIL-6R^+^ cells

**Figure 2:**
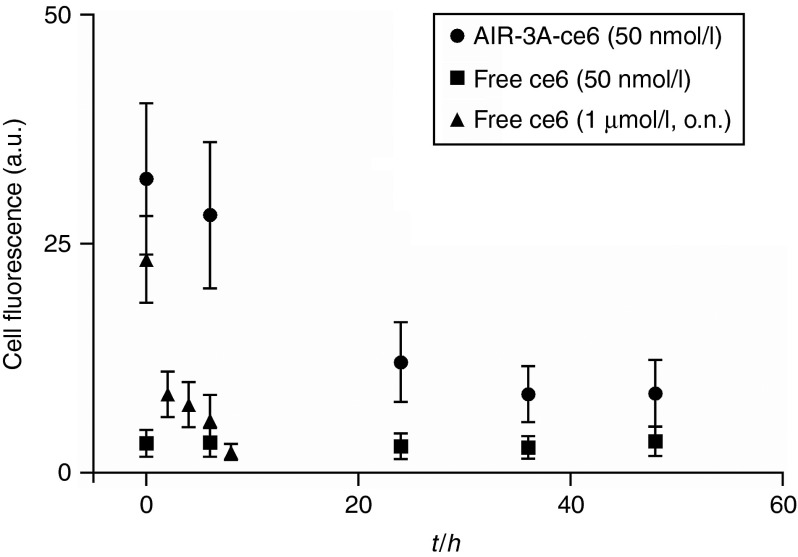
**Retention of chlorin e6 derivatized aptamer AIR-3A (AIR-3A-ce6) in BaF3/gp130/IL6R/TNF cells**. Cells were incubated with 50 nmol/l AIR-3A-ce6 or free ce6 for 45 minutes in medium without serum or with 1 µmol/l free ce6 over night. Afterwards, the cells were cultivated in serum containing medium. The retention of chlorin e6 was determined via flow cytometry. The values represent median cell fluorecence intensities ± SD.

To confirm the efficiency of aptamer-directed delivery of the photosensitizer ce6 specifically into hIL-6R^+^ cells, retention of ce6 in the cells was monitored. hIL-6R^+^ cells were exposed to 50 nmol/l of the conjugate and then allowed to rest for up to 48 hours before analyzing their ce6 fluorescence via flow cytometry (**Figure 2** and **Supplementary Figure S2a**). For all time points, strong residual fluorescence could be detected. Even 48 hours after AIR-3A-ce6 treatment, cells exhibited a ce6 fluorescence shift. Exposure of hIL-6R^+^ cells to 50 nmol/l of free ce6 did not cause any fluorescent signal in the cells (**Figure 2** and **Supplementary Figure S2b**). To yield a similar intracellular ce6 concentration compared to cells treated with AIR-3A-ce6, a 20-fold excess of free photosensitizer was needed to treat hIL-6R^+^ cells overnight (**Figure 2** and **Supplementary Figure S2c**). After media replacement, the decline of ce6 within cells was significantly faster compared to cells treated with AIR-3A-ce6. A recovery period of 8 hours was sufficient to remove all free ce6 from the cells.

**Figure 3:**
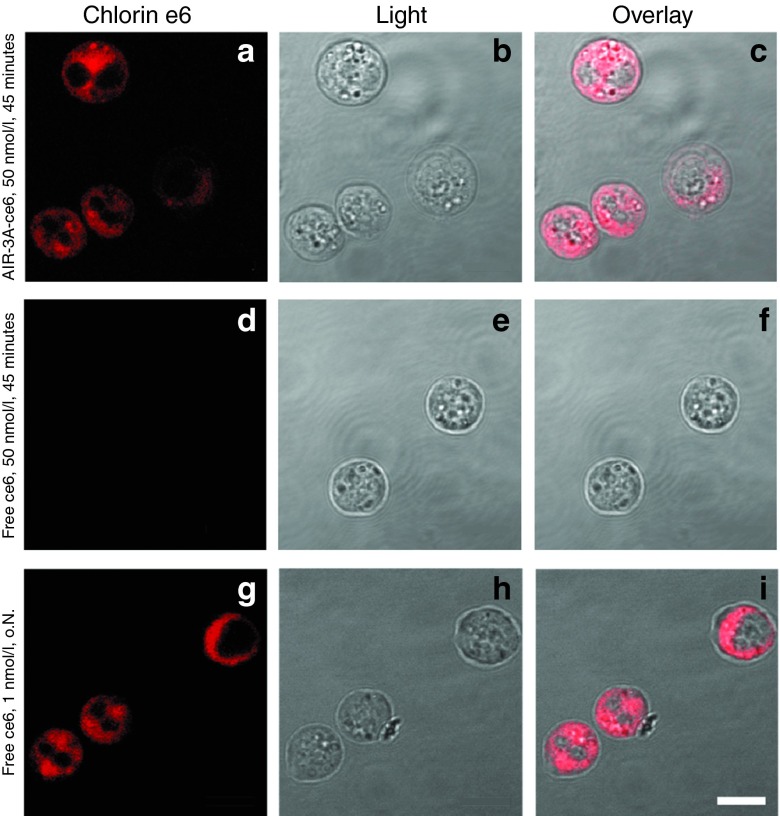
**BaF3/gp130/IL6R/TNF cells after internalization of chlorin e6 derivatized aptamer, AIR-3A-ce6**. Cells were incubated with 50 nmol/l AIR-3A-ce6 for 45 minutes in medium without serum. Accumulation of the internalized AIR-3A-ce6 was analyzed via confocal laser scanning microscopy (chlorin e6) and white light micrograph (light) confirming broad chlorin e6 distribution in the cytosol. (**a–c**) 50 nmol/l AIR-3A-ce6, 45 minutes incubation; (**d–f**) free ce6, 50 nmol/l, 45 minutes incubation; (**g–i**) over night incubation with 1 µmol/l free ce6; scalebar = 10 µm.

Subsequently, we analyzed the cellular uptake of ce6 via confocal laser scanning microscopy. When ce6 was delivered into hIL-6R^+^ cells upon incubation with AIR-3A-ce6 at a concentration of 50 nmol/l, a broad distribution of ce6 within the cytosol was detected (**Figure 3a**–**c**). In case of free ce6, no fluorescence staining of the cells could be detected at 50 nmol/l (**Figure 3d**–**f**). However, if the cells were exposed to free ce6 overnight at a concentration of 1 µmol/l, ce6 could be detected intracellularly (**Figure 3g**–**i**).

### Cytotoxicity of AIR-3A-ce6 in hIL-6R^+^ cells

**Figure 4:**
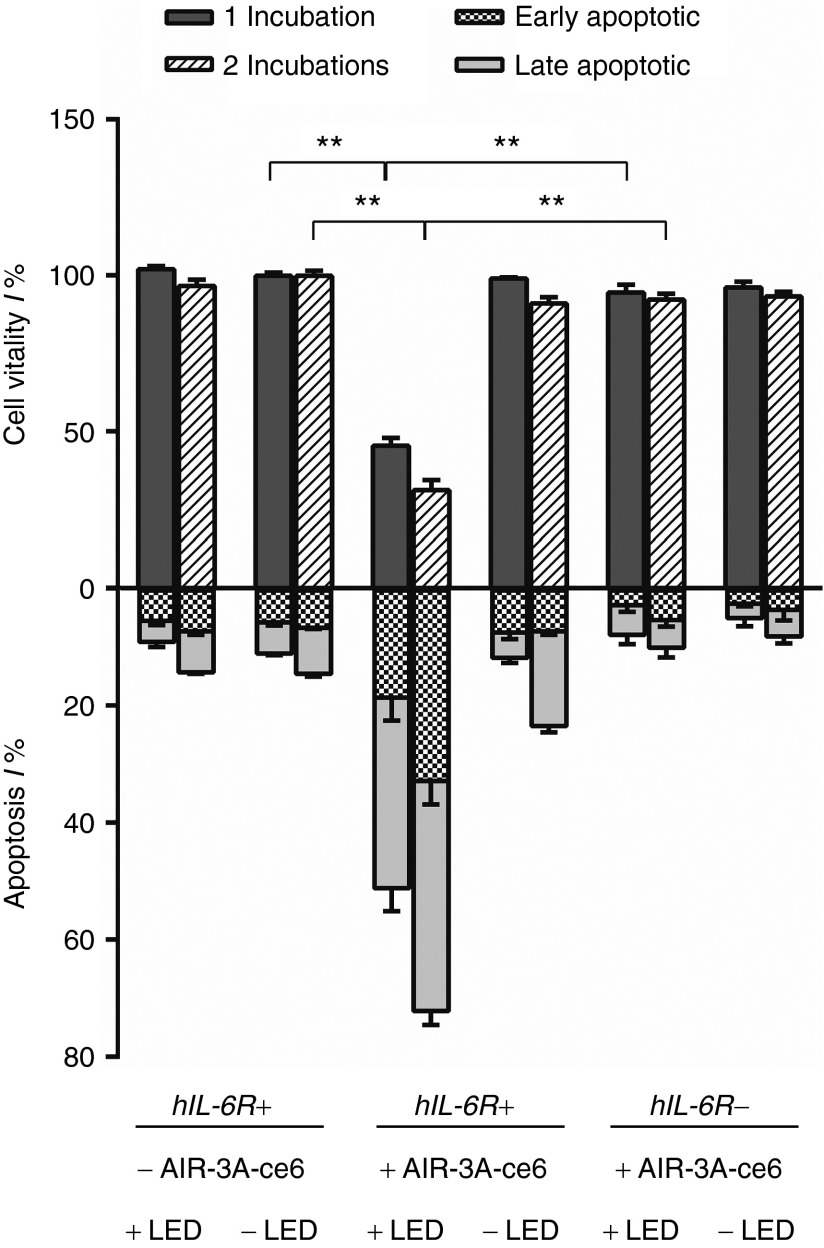
**Vitality of BaF3/gp130/IL6R/TNF cells after treatment with chlorin e6 derivatized aptamer, AIR-3A-ce6**. Cells were incubated with 150 nmol/l AIR-3A-ce6 conjugate for 25 minutes in phosphate-buffered saline containing 0.5% bovine serum albumin. Cells subjected to two incubations were rested for 3 hours between the incubations. Subsequently, the cells were irradiated (660 nm, 100 J/cm^2^; 30 mW/cm^2^) or kept in the dark. Error bars represent mean ± SD of two independently repeated experiments with one exposition (grey) or two subsequent expositions (shaded) to the aptamer-ce6 conjugate. Cell vitality values are given in reference to the control cells. Asterisks indicate significance *P* < 0.01.

After specific and efficient internalization of AIR-3A-ce6 into hIL-6R^+^ cells, we illuminated these cells with red light (660 nm) to activate the cytotoxic effects of ce6. This led to a decrease in cell viability to 45.6 ± 1.8% compared to the untreated cells (**Figure 4**). We quantified the amount of apoptotic cells via propidium iodide/annexin V-FITC staining. This method yielded a relative rate of 17.8 ± 2.3% and 41.1 ± 1.2% of early and late apoptotic cells, respectively (**Figure 4** and **Supplementary Figure S3**). If the cells were exposed twice to the AIR-3A-ce6 conjugate prior to irradiation, cytotoxicity could be further enhanced with 31.4 ± 2.3% cell survival (32.6 ± 2.8% early apoptosis, 39.3 ± 1.7% late apoptosis; **Figure 4** and **Supplementary Figure S3**). If cells were exposed to AIR-3A-ce6 without subsequent light irradiation or solely subjected to light irradiation, no cytotoxic effects could be detected. LED irradiation of AIR-3A-ce6 untreated cells did not show any effect on cell viability (+AIR-3A-ce6 –LED: 99.2 ± 0.6%; −AIR-3A-ce6 +LED: 102.2 ± 1.4%; **Figure 4** and **Supplementary Figure S3**). As expected, the illumination of control cells showed no significant effect on apoptosis (+AIR-3A-ce6 –LED: 7.2 ± 0.8% early apoptosis, 4.3 ± 0.6% late apoptosis; –AIR-3A-ce6 +LED: 5.2 ± 0.5% early apoptosis, 3.6 ± 0.6% late apoptosis; **Figure 4** and **Supplementary Figure S3**).

## Discussion

PDT has become a regular method in the treatment of human diseases, *e.g.*, cancer or inflammatory disorders. PDT agents are usually locally injected and subsequently activated by light exposure. Several approaches have been tested to increase the efficacy and specificity of PDT.^21,22^ Due to the limited migration range of singlet oxygen in the cytosol (<0.01 µm),^23^ cytotoxicity of the PDT agent is highly related to its localization within the cell.^24^ Therefore, PDT agents that are not taken up by cells, *e.g.*, uroporphyrin, are extremely inefficient despite their production of singlet oxygen.^25^

The cellular uptake and the localization of photosensitizers have been investigated in connection with several classes of carrier molecules, including polysterene microspheres,^26^ nano particles,^27^ and liposomes.^28^ Even caged PDT agents have been studied.^29^ Antibody conjugates of photosensitizers have been investigated for cell-specific delivery in a broad variety of tumors.^30,31,32^ Though some of them exhibited enhanced efficacy over the free nonspecificacting PDT agent, the preparation of those conjugates is costly, and the required chemical conjugation reactions are usually inefficient.

One promising alternative strategy is the use of aptamers as vehicles for delivering photosensitizers. The advantages of aptamers over antibody conjugates include economical solid phase synthesis, straightforward chemical modification, stability at room temperature storage, as well as nonimmunogenicity.

We examined whether delivery of PDT agents into lymphoma cells could be accomplished by RME when a receptor-specific aptamer conjugated to an appropriate photosensitizer is used. As a model system, we chose an aptamer (AIR-3A) that specifically binds to the hIL-6R of the murine pre-B cell line BaF3/gp130/hIL6R/TNF. The analogous cell line BaF3/gp130 lacking hIL-6R served as control.

We confirmed specific uptake of the aptamer conjugated to the photosensitizer chlorin e6 (AIR-3A-ce6) by hIL6R^+^ cells. 50 nmol/l of AIR-3A-ce6 were sufficient for substantial uptake within 45 minutes, while 50 nmol/l of the free ce6 were not internalized by the cells to any detectable extent in the same amount of time. Flow cytometry and confocal laser microscopy confirmed that the amount of ce6 inside the cells was significantly higher (20 times) when delivered as the aptamer conjugate when compared to the free photosensitizer treatment. Aptamer-delivered ce6 could be localized within the cytosol and was particularly associated with the nuclear membrane. Similar results could be observed for free ce6 when the cells were exposed to the photosensitizer overnight, indicating nonspecific ce6 uptake at relatively high concentrations due to its poor membrane permeability.^33^ When cells were exposed for only 45 minutes, ce6 remained anchored in the cytoplasmic membrane and therefore could rapidly be washed off upon recultivation in drug-free medium. In the case of aptamer-mediated delivery, fluorescing ce6 remained within the cells and was detectable even 48 hours after drug exposure. This is probably due to an active transport via RME.

With subsequent exposures to the aptamer conjugate, the amount of ce6 in the cells increased. This observation may benefit future RME-mediated therapies for enhancing the cytotoxic effects of certain cutaneous lymphoma cells.

**Figure 5:**
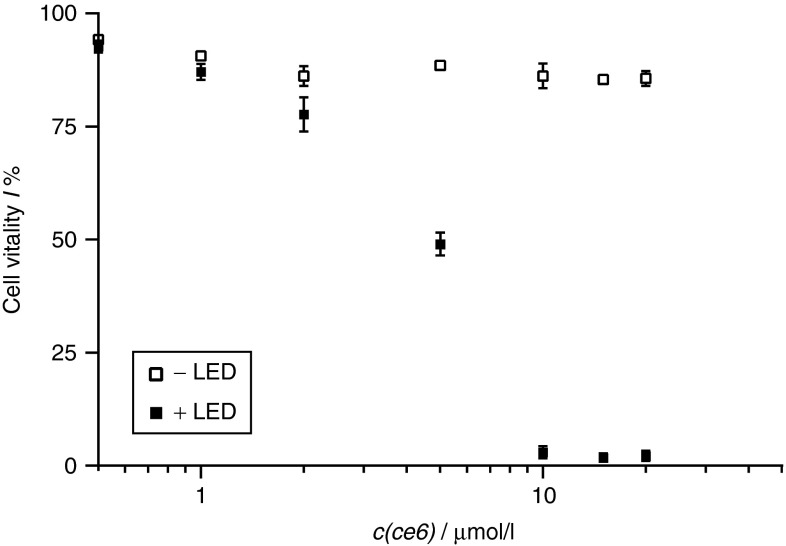
**Vitality of BaF3/gp130/IL6R/TNF cells after treatment with free chlorin e6**. Cells were incubated with ce6 over night, washed and cultivated in drug-free medium. Subsequently, the cells were irradiated (660 nm, 100 J/cm^2^; 30 mW/cm^2^) or kept in the dark. The cell viability was determined usind annexin pi double staining.

The cytotoxicity of the aptamer-delivered ce6 was confirmed upon irradiation with the typical light dose and excitation wavelength for ce6, resulting in a reduction of the cell viability to 46%. The cytotoxic effect was enhanced (31% vital cells) for cells that had been exposed to the conjugate twice and exhibited 33% apoptotic cells. To achieve comparable cytotoxicity with the same irradiation dose, micromolar concentrations of ce6 and extensions of the incubation time to several hours were needed (**Figure 5**).^34^ Hence, AIR-3A-ce6 enhanced the PDT cytotoxicity in addition to its selectivity for hIL-6R^+^ cells.

**Figure 6:**
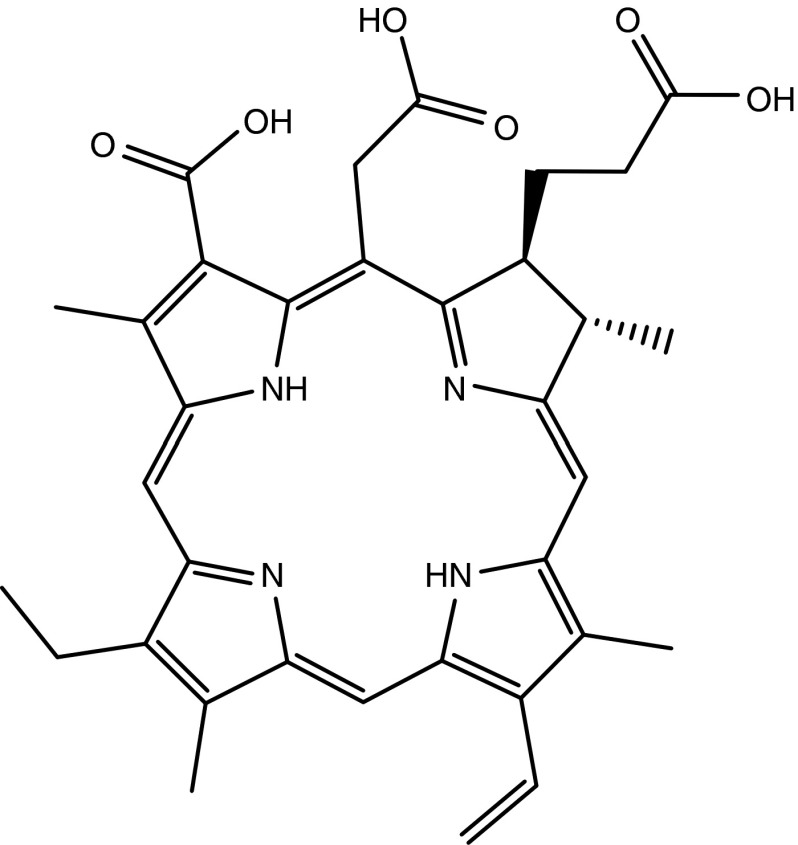
**Structure of chlorin e6**.

The here used aptamer photosensitizer conjugate fulfils all requirements for a selective drug delivery including endosomal escape of the drug due to the structural properties of ce6. Ce6 is an amphiphilic compound comprised of three adjacent carboxyl groups on a lipophilic aromatic system (**Figure 6**). At neutral pH, ce6 distributes extremely slowly via a flip-flop mechanism across lipid bilayers. Thus, it accumulates on the outer monolayer and is only taken up randomly via endocytotic events.^35^ The direct transfer of ce6 across lipid bilayers is strongly accelerated under mild acidic conditions.^36^ We assume that ce6 benefits from this fact to undergo an endosomal escape in the case of RME of AIR-3A-ce6 with IL-6R. Upon a receptor recycling of hIL-6R, lysosomal degradation of the carrier RNA (AIR-3A) and, due to endosomal acidification, protonation of the three ce6 carboxylic residues would occur (pKa range between 6.5 and 8.5). The ce6 molecule would thereby gain lipohilicity and cross the endosomal membrane rapidly into the cytosol.

To the best of our knowledge, we report the first aptamer-mediated PDT-targeting hIL6R, which is presented on cells such as those associated with cutaneous lymphoma. These cells would then be accessible to PDT via irradiation.

Other aptamers that have been reported to deliver covalently bound PDT agents are the aptamer TD05,^37^ a Ramos cells recognizing aptamer, and different Mucin-binding DNA aptamers.^38^ The aptamer TD05 was exclusively selected for Burkitt's lymphoma cells by cell-SELEX. The Mucin aptamers are binding to short O-glycan-peptides (MUC1) on the surface of breast, colon, lung, ovarian, and pancreatic cancer cells. For the latter aptamers, internalization and reactive oxygen species generation was confirmed. In that study, a recycling process of MUC1 through the trans-Golgi network, endosomal, and lysosomal compartments was harnessed for the drug delivery. The required concentration for the induction of cell death is in the same range for the MUC1 aptamer as for the IL-6R aptamer when compared to the drug only control. Our results, therefore, expand the range of aptamer-mediated chlorin e6 delivery to the field of cytokine receptors and point out the endosomal escape ability of ce6.

Since malignant tissue is not always accessible to light irradiation, PDT is either limited to cutaneous diseases or needs the surgical exposure of the affected inner organs. The aptamer photosensitizer conjugate described in this study could possibly target a broad range of malignant cell types that are accessible to the irradiation of PDT without prior surgical action, as the aptamer target IL6R is present on B- and T-lymphoma cells. Therefore, we are currently investigating the aptamer-mediated delivery of chlorin e6 to cell lines endogenously presenting IL-6R. So far, we were not able to detect a similar cytotoxic effect in these cell lines. In a broad range of diseases related to enhanced proliferation of IL-6R–positive cells, the plasma concentration of IL-6 is elevated leading to an enhanced production of IL-6R^39^ and its increased transport to the lysosomes.^40^ Those cells might be appropriate for AIR-3A-ce6–mediated PDT. An alternative might be the application of aptamers containing multiple photosensitizer units. IL-6R–related diseases like mycosis fungoides, castleman's disease, as well as inflammatory disorders such as rheumatoid arthritis and other cutaneous diseases are currently of interest for PDT and their treatment might be improved by further development of aptamer conjugates in the future.^41,42,43^

## Materials and methods

*Chemicals.* Unless otherwise noted, all chemicals were purchased from Sigma-Aldrich (Hamburg, Germany). Annexin V-FITC was purchased from Invitrogen (Karlsruhe, Germany). Buffers were prepared using deionized water obtained from a water purification system (Millipore, Billerica, MA).

*Oligonucleotides.* All RNAs were synthesized, modified, and purified by IBA Life Sciences (Goettingen, Germany).


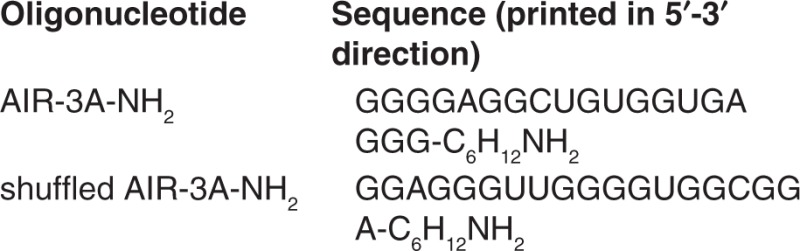


*Chlorin e6 coupling to AIR-3A and shuffled AIR-3A.* Ten mocroliter of 50 mmol/l *N*-Hydroxysuccinimide in 0.1 mol/l MES buffer (pH 6.0) were added to 10 µl of 50 mmol/l chlorin e6 (Santa Cruz Biotechnology, Santa Cruz, CA) in dimethyl sulfoxide (Carl Roth, Karlsruhe, Germany). Subsequently, 10 µl of 100 mmol/l 1-ethyl-3-(3-dimethylaminopropyl) carbodiimide were added and mixed thoroughly. The amino modified RNA (110 µl, in 0.1 mol/l phosphate buffer, pH 7.4) was added immediately; the solution was mixed by pipetting and kept at 37 °C for 1 hour. The excess of free chlorin e6 was removed by at least three subsequent ethanol precipitations. Dissolving the first precipitate in MES buffer (0.1 mol/l, pH 6.0), strongly enhanced the solubility of free chlorin e6 for subsequent removal. Gel analysis confirmed quantitative coupling reaction and total removal of free chlorin e6. Overall yield after purification was 62% with respect to the used RNA. Quantification of AIR-3A-ce6 for cytotoxicity assays was carried out by fluorescence detection on a VersaDoc Imaging System (Biorad Laboratories, Munich, Germany) using different defined amounts of chlorin e6 as a calibration standard.

*Cell cultures.* BaF3/gp130/IL6R/TNF and BaF3/gp130 cells were kindly provided by Athena Chalaris (Rose-John lab, University of Kiel, Germany). Those were cultured at 37 °C and 5% CO_2_ in Dulbecco's modified Eagle medium (PAA, E15-810) supplemented with 10% fetal bovine serum (PAA, K41-001), penicillin (60 mg/l, PAA, P11-010) and streptomycin (100 mg/l, PAA, P11-010). The culture medium for BaF3/gp130/IL6R/TNF was further supplemented with human IL-6 (10 ng/ml, PeproTech, Hamburg, Germany), whereas the culture medium for BaF3/gp130 was further supplemented with murine IL-3 (10 ng/ml, Biozol, Munich, Germany). The cells were passaged every 3 days and cultured in a density between 1 × 10^5^ and 1 × 10^6^ cells per ml. For cytotoxicity tests, BaF3/gp130 cells were also cultured in medium supplemented with Hyper-IL-6 (10 ng/ml)^44^ or human IL-6 and human sIL-6R (10 ng/ml each).

*Flow cytometry.* The presentation of human IL-6R and human gp130 on the surface of BAF3/gp130/IL6R/TNF or BAF3/gp130 cells was established by flow cytometry using antibodies specific for human IL-6R and human gp130, respectively. 5 × 10^5^ cells were washed twice in phosphate-buffered saline (PBS) and suspended in 350 µl PBS. After addition of a murine primary antibody binding to human IL-6R (B-R6, antibodies-online, ABIN123898) or human gp130 (R&D systems, Wiesbaden, Germany, MAB228), respectively, or an isotype-specific control antibody (Tetra-His antibody, Qiagen, Hilden, Germany, 34670) in final concentrations of 0.3 ng/µl, cells were incubated for 30 minutes on ice. Three washing steps with 350 µl PBS followed. Cells were incubated with an allophycocyanin (APC)- or fluoresceine isothiocyanate (FITC)-labeled secondary antibody (1:350 diluted; Th. Geyer, 550826, or Santa Cruz Biotechnology, sc-2078, respectively) in 350 µl 1× selection buffer for 30 minutes at 4 °C and then washed as previously described and resuspended in 350 µl 1× selection buffer. Fluorescence intensities were determined by a FACS Calibur flow cytometer (BD Biosciences, San Jose, CA) counting 10,000 events and evaluated using the BD CellQuest software (Version 3.2.1).

Binding to and uptake of AIR-3A-ce6, shuffled-AIR-3A-ce6 or free chlorin e6 by BaF3 cells was also determined by flow cytometry. The RNA conjugates or the free photosensitizer (50 nmol/l each) were incubated with 5 × 10^5^ to 1 × 10^6^ cells in serum lacking medium for 45 minutes at 37 °C. Afterwards, cells were washed, suspended in 350 µl of the same buffer, and analyzed by flow cytometry as described above or resuspended in serum containing medium and cultured at 37 °C and 5% CO_2_ for later repetition of incubations with the RNA-ce6 conjugates (see below cytotoxicity assays).

Binding and uptake of free chlorin e6 was also analyzed for prolonged incubation times. Therefore, defined amounts of the photosensitizer were added to the cell culture vessel overnight.

*Confocal laser scanning microscopy.* To visualize the internalization and accumulation of chlorin e6 in BAF/gp130/IL6R/TNF-cells, 5 × 10^5^ cells were incubated as described above. Cells were washed two times with 350 µl PBS and resuspended in 50 µl of the same buffer.

Suspensions were directly placed on a glass slide and covered by a cover slip. Samples were imaged with LSM 510 ConfoCor2 system (Carl Zeiss, Jena, Germany) with the following basic adjustments: HeNe-Laser (633 nm), 5% laser power, 92–246 µm pinhole diameter, beam splitters: HFT 514/633 nm and NFT 545 nm, LP 650 nm filter.

*Cytotoxicity assays.* The uptake of AIR-3A-ce6, shuffled-AIR-3A-ce6 or free chlorin e6 by BaF3 cells was conducted as described above for the binding analyses. Overall, the cells were subjected to three incubation phases with 50 nmol/l of the RNA photosensitizer conjugate or free ce6 in serum lacking medium for 45 minutes at 37 °C. After each incubation phase, the cells were washed and cultivated in serum containing medium for 5 hours. Culturing after the last incubation phase was shortened to 1 hour and followed by exposure to LED illumination (660 nm, 90 J/cm^2^ at a rate of 30 mW/cm^2^). Control cells were kept in the dark. Afterwards, the cells were analyzed by cell cytometry using propidium iodide (1 µg/ml) and annexin V-FITC. Therefore, cells were washed and resuspended in 100 µl annexin-binding buffer (10 mmol/l HEPES, 140 mmol/l NaCl, 2.5 mmol/l CaCl_2_, pH 7.4). Annexin V-FITC and propidium iodide (1 µg/ml) was added, and the cells were incubated for 15 minutes at room temperature. After the incubation, 400 µl annexin-binding buffer was added; the suspension was mixed gently and kept on ice until flow cytometry analysis.

*Statistics.* All statistical analyses were performed using Graphpad Prism 6. All values are expressed as means with standard deviations. Comparisons between two groups were done by a two-tailed unpaired *t*-test. Differences between two means with *P* < 0.05 were considered significant, *P* < 0.01 very significant and *P* < 0.001 extremely significant.

**SUPPLEMENTARY MATERIAL** **Figure S1.** Gel analysis of chlorin e6 derivatized aptamer AIR-3A (AIR-3A-ce6). **Figure S2.** Retention of chlorin e6 derivatized aptamer AIR-3A (AIR-3A-ce6) in BaF3/gp130/IL6R/TNF cells. **Figure S3.** Vitality of BaF3/gp130/IL6R/TNF cells.
