# Supplementary material for: Chlorin e6 Conjugated Interleukin-6 Receptor Aptamers Selectively Kill Target Cells Upon Irradiation
Source: Mol Ther Nucleic Acids. 2014 Jan 21;3(1):e143–. doi: 10.1038/mtna.2013.70 (PMC3910004; doi:10.1038/mtna.2013.70)
Supplement: Supplementary Information [file mtna201370x1.doc]

Supplementary Material to:

**Chlorin e6 conjugated interleukin-6 receptor aptamers selectively kill target cells upon irradiation**

Sven Kruspe^1^, Cindy Meyer^2^ and Ulrich Hahn^1^*

^1^ Chemistry Department, MIN-Faculty, Institute for Biochemistry and Molecular Biology, Martin-Luther-King-Platz 6, 20146 Hamburg, Germany

^2^ Howard Hughes Medical Institute, Laboratory of RNA Molecular Biology, The Rockefeller University, 1230 York Ave, New York, NY 10065, USA

* To whom correspondence should be addressed.

Tel: +49 40 42838 3214

Fax: +49 40 42838 2848

Email: uli.hahn@uni-hamburg.de

**
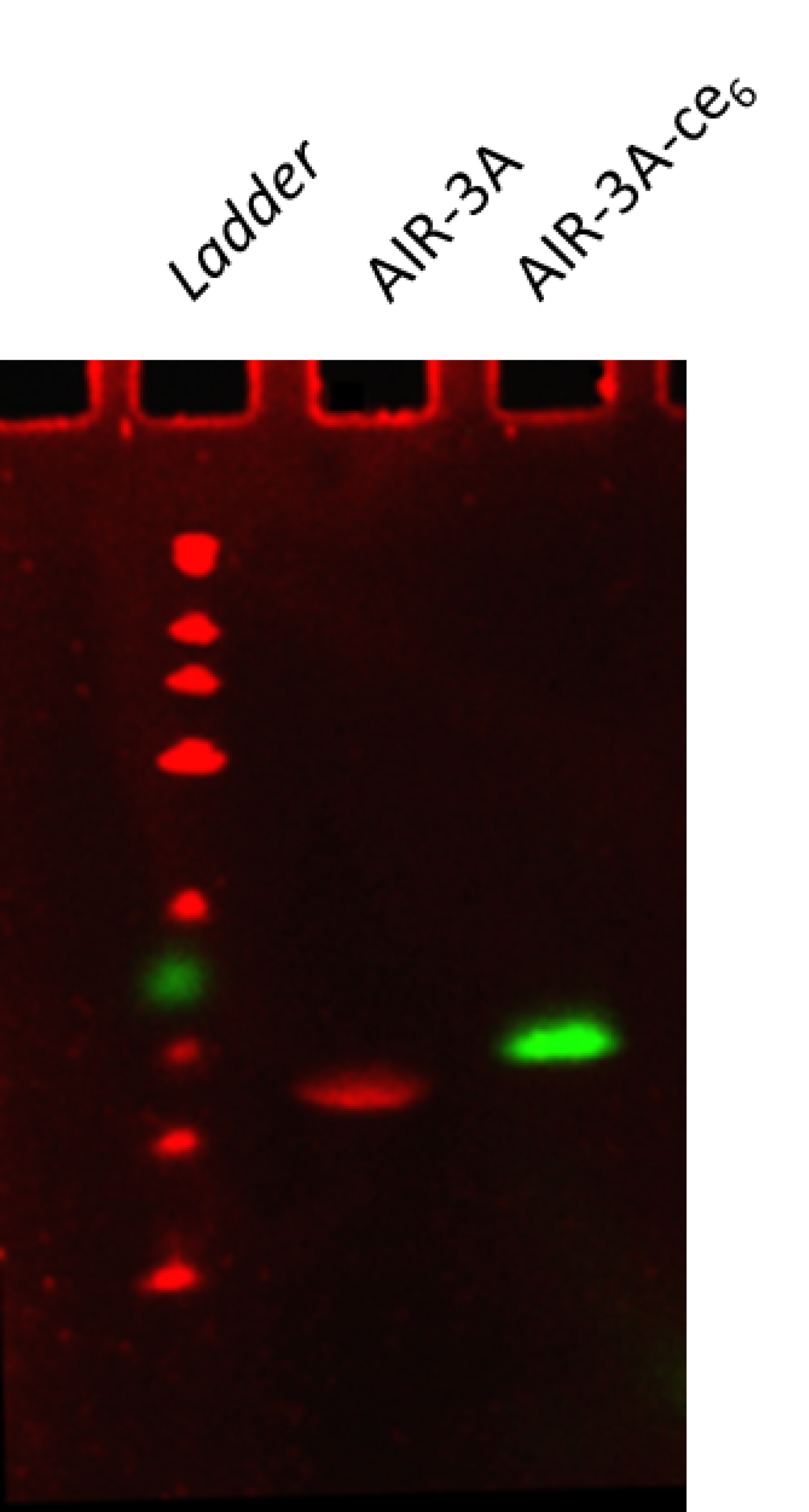
**

**Figure S1 Gel analysis of chlorin e6 derivatised aptamer AIR-3A (AIR-3A-ce6)**. Educt and product oligonucleotides of the chlorin e6 coupling were analysed via PAGE (15%) containing 8 M urea; red, EtBr stain; green, fluorescence at 695 nm.


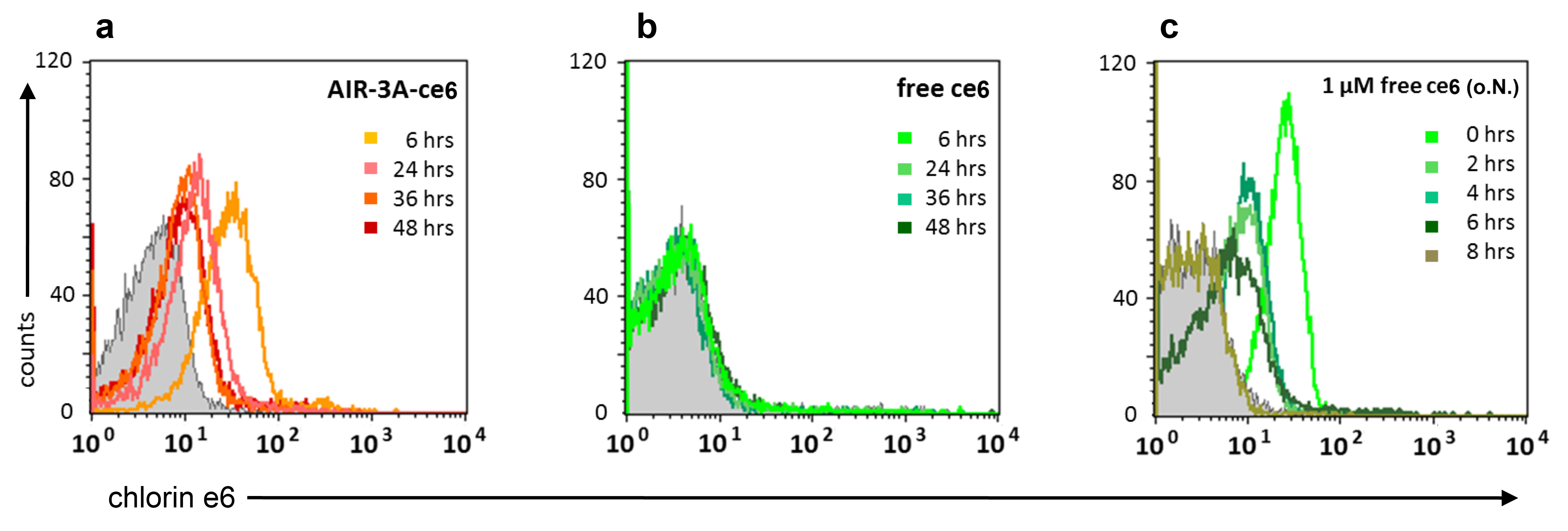


**Figure S2 Retention of chlorin e6 derivatised aptamer AIR-3A (AIR-3A-ce6) in BaF3/gp130/IL6R/TNF cells.** Cells were incubated with 50 nM AIR-3A-ce6 conjugate for 45 min (a) or free ce6 (b) in medium without serum. Afterwards the cells were cultivated in serum containing medium. The retention of ce6 was determined via flow cytometry after 6, 24, 36 and 48 h. As free ce6 did not exhibit a substantial accumulation under these conditions, cells were also exposed to 1 µM free ce6 over night (c) and the decay of the ce6 fluorescence was measured after 0-8 h.

**
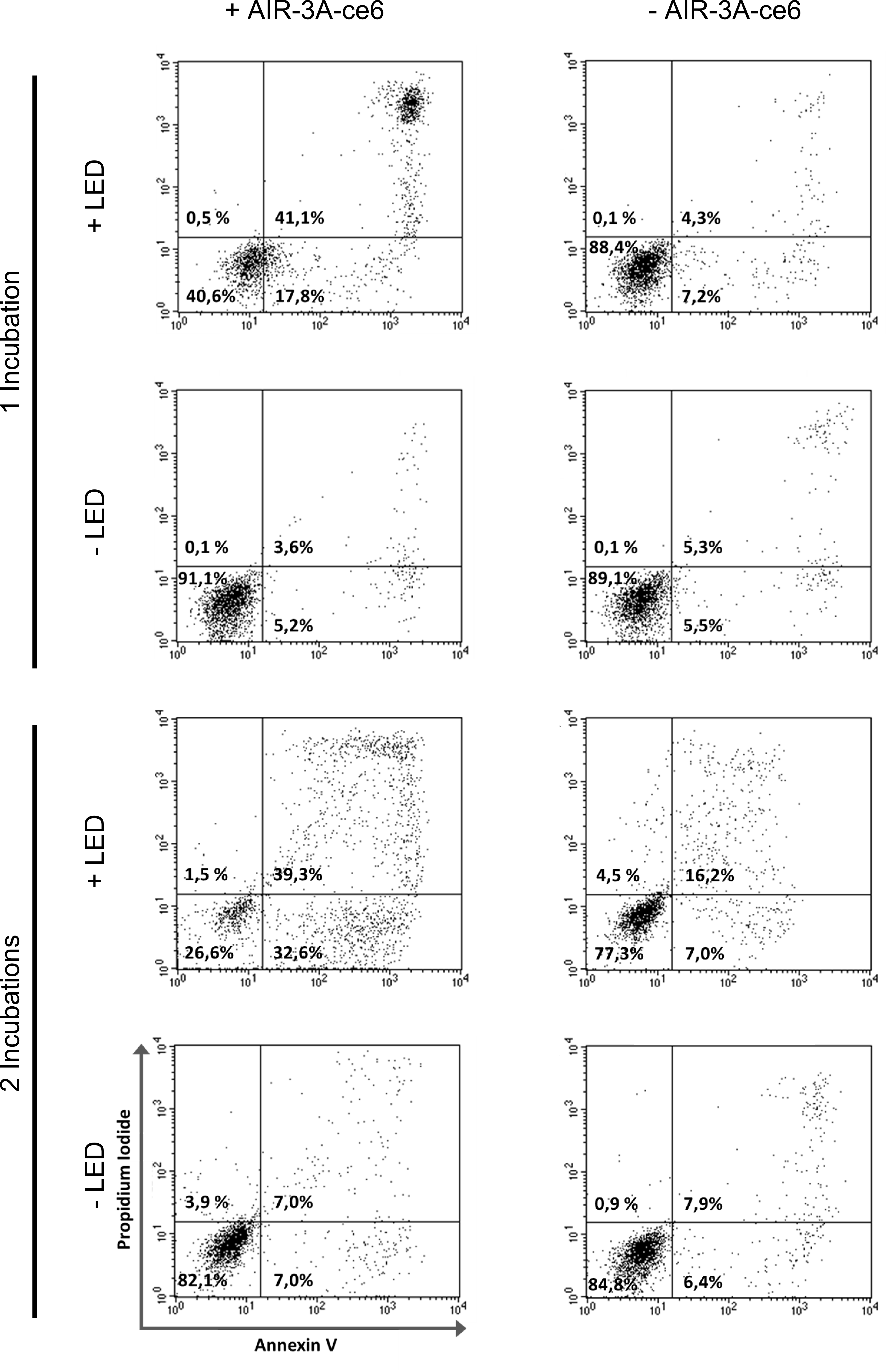
**

**Figure S3 Vitality of BaF3/gp130/IL6R/TNF cells.** Cells were incubated with 150 nM AIR-3A-ce6 conjugate for 25 min in PBS containing 0.5% BSA. Between exposures to AIR-3A-ce6 cells were cultured in medium containing serum for 3 h. Cell vitality was determined via flow cytometry using propidium iodide/Annexin V-FITC staining. Vital cells appeared in the lower left square, apoptotic cells right shifted and dead cells high shifted.
